# Supplementary material for: Raman evidence for pressure-induced formation of diamondene
Source: Nat Commun. 2017 Jul 21;8:96. doi: 10.1038/s41467-017-00149-8 (PMC5522393; doi:10.1038/s41467-017-00149-8)
Supplement: Supplementary file 1 — Supplementary Information [file 41467_2017_149_MOESM1_ESM.pdf]

File name: Supplementary Information
Description: Supplementary Figures, Supplementary Tables, Supplementary Notes and Supplementary References

File name: Peer Review File
Description:

Supplementary Note 1: Hydrostaticity of the water medium

The hydrostaticity of the water medium was monitored in our experiments by analyzing the separation between the R_1 and R_2 fluorescence lines (R_1 - R_2) from the ruby crystal, and the full width at half maximum of the R_1 line (Γ_{R1}). The data (see Supplementary Figure 1 below with (a,b) and (c,d) obtained for (I) and (II) G/G/T-H_2O experiments, respectively) suggest that the water medium is hydrostatic between 1-2 GPa, quasi-hydrostatic between 2 and 10 GPa, and non-hydrostatic above 10 GPa. This observation is supported by previous results of high-pressure experiments with water.[1] Therefore, the broadening of the G band in Figures 1(b,c) could, in fact, be assigned to the quasi-hydrostaticity of the water medium. However, besides the loss of hydrostaticity of the medium, the confinement of the E_{2g} phonons within the sp^2 domains after the diamondene formation also generates G band broadening, and cannot be ruled out in the experiments using water as PTM.

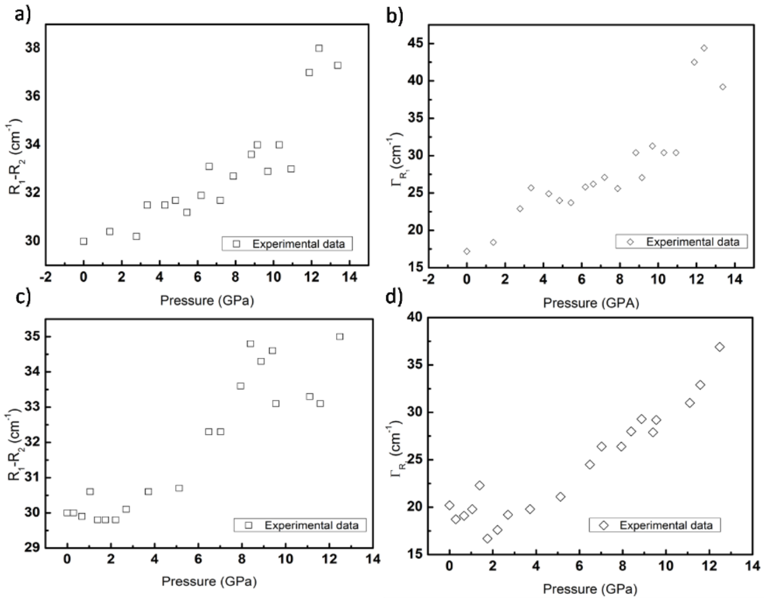

Supplementary Figure 1. Hydrostaticity of the water medium. R_1 - R_2 [panels (a) and (c)] is the separation between the R_1 and R_2 fluorescence lines (R_1 - R_2) from the ruby crystal, and Γ_{R1} [panels (b) and (d)] is the full width at half maximum of the R_1 line (Γ_{R1}). (a,b) and (c,d) were obtained in the experiments performed in samples (I) and (II) G/G/T-H_2O, respectively

Nevertheless, our main experimental result, which is the abrupt change in $\Delta\omega_G$ for the samples (I) and (II) G/G/T-H2O, has no connection with the quasi- or non-hidrostatitcity of the PTM because the spectra obtained for both blue and green lasers were always measured at the same spot in the sample for each pressure. Therefore, although the quasi- or non-hydrostaticity of the PTM can cause some stress and non-uniformities across the graphene sample, it cannot cause any change in $\Delta\omega_G$.

Supplementary Note 2: Step-function fitting and statistical analysis of the $\Delta\omega_G$ X P data

Even though there are fluctuations in $\Delta\omega_G$ over the whole pressure range, the data obtained in the 1st run can be grouped into two sets with distinct mean values [Fig 2(c)], with, in each group, fluctuations occurring about the mean. For the 2nd run [Figure 2(f)], there are three distinct groups. We have analyzed the $\Delta\omega_G$ x P data extracted from the two G/G/T-H_2O samples [(I) 1st and (II) 2nd runs summarized in Figures 2(c) and 2(f), respectively] by fitting them with step functions using *MATLAB®*. Besides that, we performed a Hypothesis Test on the difference in means for unknown variances using the statistics software *R*. Finally, we applied the same procedures to analyze $\Delta\omega_G$ x P data extracted from the G/G/T-Nujol and G/T-H_2O experiments [Figures 6(b) and 6(e), respectively]. As shown in the next lines, the occurrence of distinct values of $\Delta\omega_G$ in these two data sets is not statistically supported.

Supplementary Table 1 gives the information extracted from the fit of the $\Delta\omega_G$ x P data obtained from samples (I) and (II) G/G/T-H_2O. Here *a*, *b* and *c* are the fitting parameters (constant $\Delta\omega_G$ values in a well-defined plateau), P is the independent variable (pressure), and R^2 is, as usual, the coefficient of determination. We fixed the critical pressures separating discontinuities in the $\Delta\omega_G$ x P data: 7.5 GPa for the sample (I) G/G/T-H_2O; 5 and 10 GPa for sample (II) G/G/T-H_2O. The values of the fitting parameters, with 95% confidence bounds shown in parenthesis, are given in Supplementary Table 1. From these parameters, it is possible to infer that the $\Delta\omega_G$ x P data for the G/G/T-H_2O samples are well explained by a Step-Function model. The same cannot be said about the data from the G/G/T-Nujol and G/T-H_2O samples, for which a critical pressure value could not be defined.

Sample	Adjusted function	*a*	*b*	*c*	R^2
(I) G/G/T/H_2O	*a* if P< 7.5<br>*b* otherwise	0.51<br>(0.04, 0.98)	3.92<br>(3.43, 4.42)	-	0.87
(II) G/G/T/H_2O	*a* if P< 5<br>*b* if 5≤P<10<br>*c* otherwise	-0.13<br>(-0.55, 0.29)	2.67<br>(2.15, 3.19)	6.67<br>(5.97, 7.43)	0.95

Supplementary Table 1. Fitting parameters. Information extracted from the fit of the $\Delta\omega_G$ x P data obtained from samples (I) and (II) G/G/T-H_2O. *a*, *b* and *c* are the fitting parameters (constant $\Delta\omega_G$ values in a well-defined plateau), P is the independent variable (pressure), and R^2 is the coefficient of determination.

To further detect a statistically significant variation between different groups of data for a given sample, a Hypothesis Test on the difference in means for unknown variances was performed using the statistics software ***R***. For the (I) G/G/T/H_2O sample, the $\Delta\omega_G$ data were separated into two groups: below ($\Delta\omega_{G,1}$) and above ($\Delta\omega_{G,2}$) the critical pressure $P_{c\ 1,2}$. For the sample (II) G/G/T/H_2O, the data were divided into three groups: $\Delta\omega_{G,1}$ and $\Delta\omega_{G,2}$ and $\Delta\omega_{G,3}$, defined by the two critical pressures $P_{c\ 1,2}$ and $P_{c\ 1,3}$. The data extracted from the G/G/T-Nujol and G/T-H_2O samples were separated into two groups, below and above a fictitious critical pressure P^* = 6.5 GPa. These groups are exhibited in Supplementary Table 2.

Sample	$\Delta\omega_{G,1}$	$\Delta\omega_{G,2}$	$\Delta\omega_{G,3}$	P_c (GPa)
(I) G/G/T/H_2O	{0, 0, 1.5, 1.5, 0.4, 0, 1.3, -0.2, 0.9, -0.3}	{3.8, 3.5, 4.1, 2.8, 4, 4, 4, 5.4, 3.7}	-	$P_{c\ 1,2}$=7.5
(II) G/G/T/H_2O	{0, 0.3, -0.8, -0.1, -0.1, -0.3, -0.2, 0.5, -0.5}	{4.2, 2, 2.9, 3, 2.1, 1.8}	{6.6, 6.9, 6.6}	$P_{c\ 1,2}$= 5<br>$P_{c\ 1,3}$=10
G/G/T-Nujol	{0, 1.3, 0.2, 0.2, 1.2, 1.2, 0.9, 1.4, 0.8}	{0.6, -0.1, 0.7, 3.4, 0.7, 2.6, 2.5, 0.5}	-	P*=6.5
G/T-H_2O	{0, 2.1, 0.3, 1.8, 1, -0.9, 1.2}	{2.6, 1.6, 0.5, 1.2, 0.2, -1.4, 7, 2.1}	-	P*=6.5

Supplementary Table 2. Grouping the $\Delta\omega_G$ data into different sets. Information extracted from the fit of the $\Delta\omega_G$ x *P* data obtained from samples (I) G/G/T-H_2O, (II) G/G/T-H_2O, G/G/T-Nujol and G/T-H_2O. *P** is fictitious critical pressure.

The $\Delta\omega_G$ data within each group are considered as obtained from independent samples. This choice assumes that the source of change in $<\Delta\omega_G>$ is solely the pressure (the symbol <> stands for the population average). The results extracted from the Hypothesis Tests for all samples, with the Null and Alternative Hypothesis stated for each sample, are exhibited in Supplementary Table 3. There, d_f is the degree of freedom, *t*-value, *t*-critical and *p*-value have their usual meaning, and the significance is 0.05.

Sample	Null Hypothesis	Alternative Hypothesis	d_f	*t*-value, *t*-critical (0.05 significance), p-value	Accept/ Reject Null Hypothesis
(I) G/G/T/H_2O	$<\Delta\omega_{G,1}> = <\Delta\omega_{G,2}>$	$<\Delta\omega_{G,2}> > <\Delta\omega_{G,1}>$	17	10.54, 1.74, 3.514×10^{-9}	Reject
(II) G/G/T/H_2O	$<\Delta\omega_{G,1}> = <\Delta\omega_{G,2}>$	$<\Delta\omega_{G,2}> > <\Delta\omega_{G,1}>$	6	7.19, 1.9 , 1.483×10^{-4}	Reject
(II) G/G/T/H_2O	$<\Delta\omega_{G,1}> = <\Delta\omega_{G,3}>$	$<\Delta\omega_{G,3}> > <\Delta\omega_{G,1}>$	10	28.65, 1.81, 3.124×10^{-11}	Reject
G/G/T-Nujol	$<\Delta\omega_{G,1}> = <\Delta\omega_{G,2}>$	$<\Delta\omega_{G,1}> \neq <\Delta\omega_{G,2}>$	9	1.16, 2.26, 0.2743	Accept
G/T-H_2O	$<\Delta\omega_{G,1}> = <\Delta\omega_{G,2}>$	$<\Delta\omega_{G,1}> \neq <\Delta\omega_{G,2}>$	13	0.93, 2.16, 0.3683	Accept

Supplementary Table 3. Hypothesis Tests. Results extracted from the Hypothesis Tests for all samples, with the Null and Alternative Hypothesis. d_f is the degree of freedom, t-value, t-critical and p-value have their usual meaning, and the significance is 0.05.

For each Hypothesis test, the equality in the population variance of the two analyzed data sets was tested with the *F* test and, depending on the outcome, a suitable expression to obtain the *t*-value was used. For the normality analysis, we employed the Shapiro-Wilk test and an additional QQ Plot. The results of the Shapiro-Wilk test are presented in Supplementary Table 4, where *W* is the test statistics. The Null Hypothesis for the Shapiro-Wilk test corresponds to a normally distributed population with 0.05 of significance. The Shapiro-Wilk tests showed no evidence against the assumption that the $\Delta\omega_G$ distribution is normal for all samples. This conclusion is supported by visual inspection of the QQ Plots (Supplementary Figure 2).

Sample	*W*	*p*-value	Accept/Reject Null Hypothesis
(I) G/G/T/H_2O	0.95781	0.5301	Accept
(II) G/G/T/H_2O	0.93207	0.2111	Accept
G/G/T-Nujol	0.94192	0.3416	Accept
G/T-H_2O	0.88857	0.06381	Accept

Supplementary Table 4. Shapiro-Wilk test. Results of the Shapiro-Wilk test employed for the normality analysis of the $\Delta\omega_G$ distribution. *W* is the test statistics. The test shows no evidence against the assumption that the $\Delta\omega_G$ distribution is normal for all samples.

From the results of the Hypothesis Test, we reject the Null Hypothesis at 0.05 level of significance for the (I) G/G/T/H_2O and (II) G/G/T/H_2O samples, which means that the observed changes in $\Delta\omega_G$ after the critical pressures for these samples are statistically significant and cannot be attributed to chance. For the G/G/T-Nujol and G/T-H_2O samples, we accept the Null Hypothesis at 0.05 level of significance, which means that there is no statistically significant change in $\Delta\omega_G$ after the fictitious critical pressure P^*. In practice, it means that observed changes in $\Delta\omega_G$ after this pressure can be attributed to chance.

In summary, from the combined information provided by the Hypothesis Test and the step function fitting, we conclude that there is a statistically significant change in $\Delta\omega_G$ for pressures above 7.5 GPa for the sample (I) G/G/T/H_2O, and above 5 GPa for sample (II) G/G/T/H_2O. The same is not observed for the G/G/T-Nujol and G/T-H_2O samples, as expected. The statistical analysis show that the changes observed in the (I) G/G/T/H_2O and (II) G/G/T/H_2O $\Delta\omega_G$ x P data cannot be attributed to chance. Therefore, it allows inferring that the plateaus observed in Figures 1(c) and 1(f) are robust. The fluctuations in $\Delta\omega_G$ that occur before these critical pressures for the (I) G/G/T/H_2O and (II) G/G/T/H_2O samples, and for the whole pressure range for the G/G/T-Nujol and G/T-H_2O samples, could be attributed to the experimental error in determination of this variable.

(a)

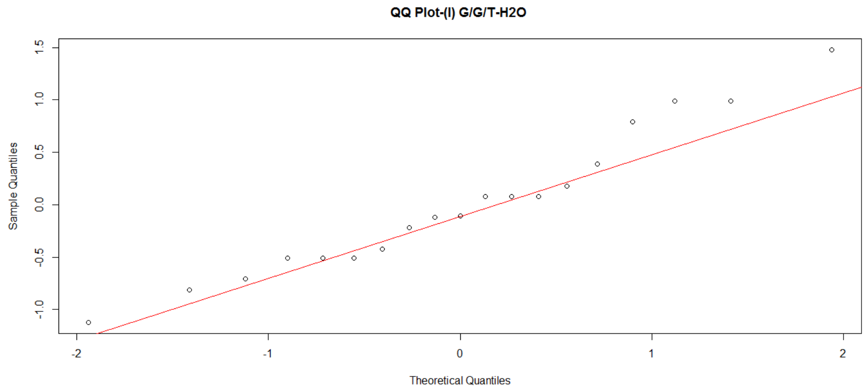

(b)

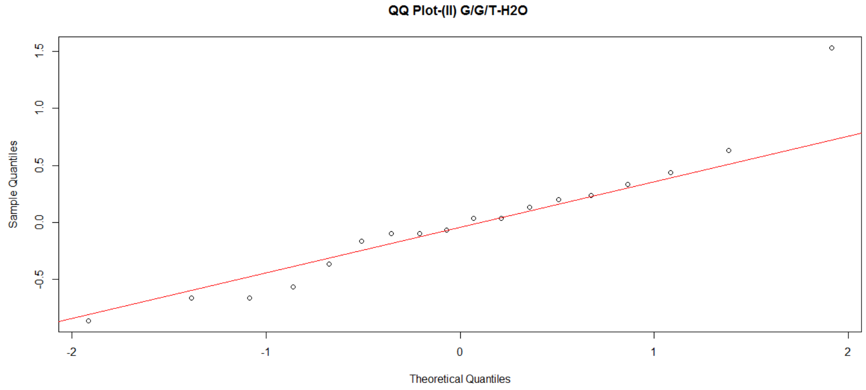

(c)

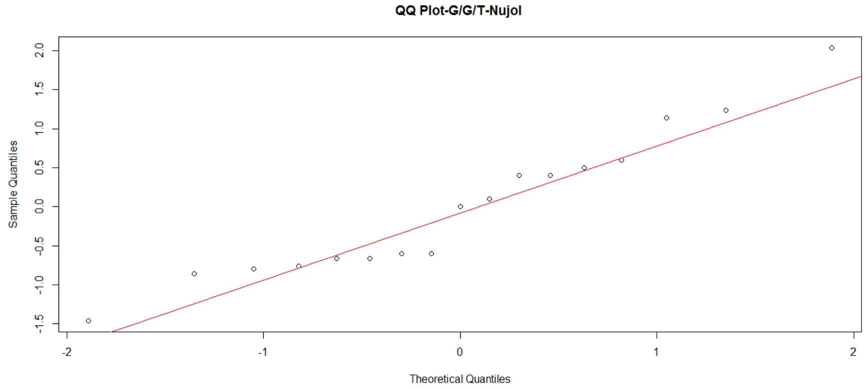

(d)

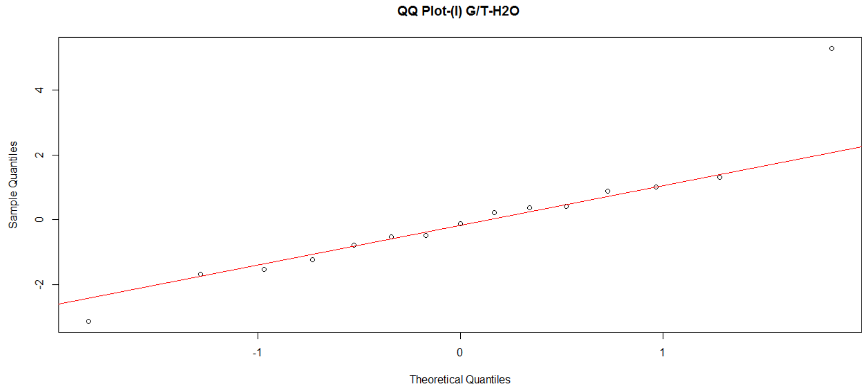

Supplementary Figure 2. QQ plots. (a) Sample (I) G/G/T-H_2O. (b) Sample (II) G/G/T-H_2O. (c) Sample G/G/T-Nujol. (d) Sample G/T-H_2O.

Supplementary References

1. G. J. Piermarini, S. Block, and J.D. Barnett. Hydrostatic limits in liquids and solids to 100 kbar. J. of Appl. Phys. **44**, 5377-5382 (1973).
